# Bacterial communities in co-cultured fish intestines and rice field soil irrigated with aquaculture wastewater

**DOI:** 10.1186/s13568-022-01475-x

**Published:** 2022-10-22

**Authors:** Weibing Guan, Kui Li, Kejun Li

**Affiliations:** College of Marine Ecology and Environment, Shanghai Ocean University, Shanghai, China

**Keywords:** Asian carp, Bacterial community, Integrated rice-fish system, Intestinal bacteria, Soil bacteria

## Abstract

In some regions, integrated rice-fish farms have been developed to balance the needs of aquaculture wastewater discharge and rice field irrigation. In this type of aqua-agriculture system, soil is irrigated with aquaculture wastewater, and intestinal bacteria in cultured fish species likely impact soil bacteria through irrigation. However, little is known about the relationship between soil bacteria and intestinal bacteria in some carp species commonly co-cultured in some Asian regions. Therefore, we co-cultured five carp species in aquaculture ponds and used the aquaculture wastewater to irrigate rice fields for over 5 years, and then compared carp intestinal bacterial communities with rice field soil bacterial communities. The results from analysis of similarity and SourceTracker analysis showed that a low similarity (R = 0.7908, P = 0.001) and contribution (an average of 9.9% of bacterial genera) of intestinal bacteria to soil bacterial communities although 77.5% of soil bacterial genera were shared by intestinal bacteria. Our results also indicated that intestinal bacteria in the numerically dominant fish species in the co-culture system do not necessarily impact soil bacteria more significantly than those of less abundant carp species, and that intestinal bacterial communities in one single fish species may impact certain soil bacterial phyla more significantly than others. Our results provide a better understanding of the impact of aquaculture wastewater on rice fields and will be helpful for the development of this type of aqua-agriculture system.

**Supplementary Information:**

The online version contains supplementary material available at 10.1186/s13568-022-01475-x.

## Introduction

Fish are an important protein resource for humans. In recent years, population growth has led to an increased demand for fish, which has resulted in overfishing and the decline of wild fish stocks (Yang et al. 2019). Aquaculture production has increased to alleviate the increasing pressure of fish demand. Zhang et al. (2022) reported that freshwater finfish culture dominates global aquaculture production, and they predicted that aquaculture will continue to depend more on land-based systems than on those in the sea.

Carp species, such as black carp (*Mylopharyngodon piceus*), grass carp (*Ctenopharyngodon idella*), silver carp (*Hypophthalmichthys molitrix*), bighead carp (*Hypophthalmichthys nobilis*), common carp (*Cyprinus carpio*), and crucian carp (*Carassius auratus*), belong to the family *Cyprinidae*. Some of these carp species have been cultured in Asia for centuries (Nakajima et al. 2019) and account for a large proportion of freshwater aquaculture production (Phelps et al. 2017; Li et al. 2021a). Traditionally, carp species are co-cultured in the same ponds to optimize the use of feed and space by integrating multiple trophic levels (Wang et al. 2016; Li et al. 2021a), and high-density co-culture of these carp species has been developed in many regions.

At the same time, concerns about the ecological consequences of aquaculture wastewater discharge are growing. Aquaculture practices frequently lead to water pollution (Grabicova et al. 2020; Han et al. 2020; Ta and Babel 2020), particularly that caused by nitrogenous compounds generated by feed and fish feces (Qi et al. 2019; Kim et al. 2020). Aquaculture wastewater discharge frequently causes eutrophication in receiving water bodies. In some regions, purification and reuse of aquaculture wastewater in aquaculture farms is mandatory and direct discharge into natural waters is prohibited.

Many techniques have been developed to combat aquaculture pollution (Zadinelo et al. 2018; Choi et al. 2020). For example, aquaculture wastewater has been used to irrigate agricultural crops in some aquaculture-agriculture complexes. Rice (*Oryza sativa*) is a major global food crop and requires a huge amount of water to grow. Approximately 30 million hectares of rice fields are cultivated in China, and the use of aquaculture wastewater in rice fields is widespread in some regions of northwest China, also partly because of limited water resources.

Soil bacteria play a crucial role in the soil ecosystem, and their diversity and community structure have been widely studied in different rice field environments (Chen et al. 2017; Huang et al. 2020; Li et al. 2021b). They transform soil structure, decompose organic matter, circulate soil nutrients (Baldrian 2019; Kumar et al. 2019; Hermans et al. 2020), and support plant growth (Garbeva et al. 2004; Tartaglia et al. 2020; Van Tung et al. 2021). Soil bacterial community structure can also be an indicator of the quality of the soil ecosystem and of the soil itself (Hermans et al. 2020). Many studies have also focused on bacterial communities in different aquaculture systems (Martins et al. 2013; Chang et al. 2019; Li et al. 2022b). Bacteria in fish intestines were found to be important in digestion and immunity of host animals (Cabello et al. 2020; Neissi et al. 2020) by producing enzymes that can decompose food and release many kinds of essential and beneficial biological substances (Rurangwa and Verdegem 2015) and by suppressing pathogen growth via antagonistic effects and mucosal protection (De Schryver and Vadstein 2014). Intestinal bacterial communities in different species of Asian carp have been reported in a number of studies (Ni et al. 2021; Yu et al. 2021).

When irrigating rice fields with aquaculture wastewater, fish feces carrying intestinal bacteria may be brought into the fields. Several researchers previously reported that fish culture and irrigation using culture wastewater significantly impacted soil bacterial communities in rice fields (Chen et al. 2017; Zhao et al. 2021). However, direct comparisons of intestinal bacterial communities in co-culture fish species and soil bacteria in rice fields irrigated with aquaculture wastewater have not been reported to date. Which fish species in the co-culture system impacted the soil bacterial communities more significantly or contributed more to the impacts of aquaculture wastewater on the soil bacterial communities? Are the dominant fish species certainly impact soil more significantly or it depends on the species-specificity? One fish species impacted all soil bacterial phyla to the same extent?

In this study, we compared intestinal bacterial communities in five co-cultured carp species with soil bacterial communities in rice fields irrigated with aquaculture wastewater for over 5 years. The results of this study can provide a better understanding of the impact of aquaculture wastewater irrigation on rice field soil in a rice-fish system.

## Materials and methods

### Experimental area

One rice-fish farm located in the northwest of China (106.36°E, 38.62°N) was used in this study. Thirty hm^2^ of aquaculture ponds were used to co-culture five Asian carp species (grass carp, common carp, silver carp, crucian carp, and bighead carp). Additionally, 50 hm^2^ of fields were used to grow rice, and they were irrigated with aquaculture wastewater for more than 5 years. The fish stocking density was 15–20 thousand individuals per hm^2^. A single artificial compound feed was used in this farm, consisting of 28% crude protein, 18% crude ash, 9% crude fiber, and 4% crude fat. In this study, we collected fish from five aquaculture ponds to obtain intestinal bacteria and took water samples from four ponds and soil samples from six rice fields to obtain water and soil bacteria. Details about the sampling sites are provided in the Supporting Information (Additional file 1: Figure S1 and Additional file 4: Table S1).

### Sample collection

Samples were collected in August of 2020. In the month of sampling, this study region is sunny and dry, and the daily maximum and minimum air temperatures are around 30 °C and 18 °C, respectively. Three individuals of each fish species from each pond were randomly chosen to collect the intestinal contents. The length, weight, and height of each fish were measured. The fish were rapidly killed and the intestinal tract from the stomach or fore-intestine to the anus, excluding the stomach or fore-intestine, was removed using a sterile surgical lancet and scissors. The intestinal contents of each fish were then squeezed into sterile 50 ml centrifuge tubes using sterile tweezers (Wu et al. 2012; Sun et al. 2020), and the wet weight of the sample was measured. After being well mixed, approximately 200 mg of the intestinal contents were placed in sterile 1.5 ml centrifuge tubes and stored at− 80 °C until subsequent analysis. A total of 69 intestinal samples were collected. All experiments involving animals were performed in accordance with the protocols approved by the Animal Ethics Committee of Shanghai Ocean University (Approval ID: SHOU-DW-2020–057).

Surface water samples at 20 cm under the water surface were collected from the central points of the aquaculture ponds twice, with a 2-week interval between sampling. Immediately after sampling, the 500 ml water samples were filtered using 5-μm pore filters to remove suspended feed, feces, and planktonic algae (Zhao et al. 2017; Liu et al. 2019). Next, 0.22-μm pore filters were used to collect planktonic bacteria samples (Li et al. 2022a). All collected planktonic bacteria samples on filters were stored in sterile centrifuge tubes at− 80 °C until subsequent analysis. A total of 8 planktonic samples were collected.

In each rice field, soil samples were collected from four evenly distributed sampling sites. At each sampling site, soil between rice plants was collected from the soil surface with a depth of 0 − 5 cm (Zhou et al. 2021). The collected soil was well mixed, and approximately 100 mg of soil were place in sterile 1.5 ml centrifuge tubes and stored at− 80 °C until subsequent analysis. A total of 24 soil samples were collected.

### Bacterial community analysis

Metagenomic DNA in bacterial communities was extracted from each collected sample using the E.Z.N.A. soil DNA Kit (Omega Bio-Tek, Norcross, GA, USA). Using the primers 338F and 806R (Srinivasan et al. 2012) with sequencing barcodes, the V3–V4 hypervariable region of the bacterial 16S rRNA gene was amplified from extracted metagenomic DNA using an Applied Biosystems GeneAmp 9700 PCR thermocycler (Carlsbad, CA, USA). PCR amplicons of the bacterial 16S rRNA gene were paired-end sequenced (2 × 300) on an Illumina MiSeq platform (San Diego, CA, USA) by a commercial company (MajorBio, Shanghai, China). Raw 16S rRNA gene sequencing reads were quality-filtered by fastp (Chen et al. 2018) and merged by FLASH (Magoč and Salzberg 2011). Operational taxonomic units (OTUs) were clustered using Uparse (Edgar 2013). Alpha diversity indexes were calculated using Mothur (Schloss et al. 2009), and beta diversity (Bray–Curtis distance) was calculated using Qiime (Bolyen et al. 2019). The taxonomy of each OTU representative sequence was analyzed by RDP Classifier (Wang et al. 2007) against the Silva v 138 16S rRNA database (Quast et al. 2013). The raw reads of 16S rRNA gene sequences obtained in this study were submitted to the NCBI SRA database under the accession number PRJNA741343.

### Statistical analysis

Statistical analysis was carried out using R version 3.3.1 (The R Foundation, Vienna, Austria). We compared the diversity indexes and bacterial abundances between sample groups using Student’s *t*-test and Helch's *t*-test (R stats package). The numbers of core bacterial OTUs were counted using Venn diagram analysis (R Venndiagram package). The differences in community structure between sample groups were detected using analysis of similarities, non-metric multidimensional scaling analysis, and hierarchical clustering community heatmaps (R vegan package). In addition, we used SourceTracker (Knights et al. 2011) to analyze the contribution of intestinal bacteria to soil bacterial communities. Unless otherwise stated, all analyses were performed at the OTU level.

## Results

### Bacterial communities

The measured body parameters of 69 fish individuals and the alpha diversity data for all 101 intestinal, planktonic and soil samples are provided in the Supporting Information (Additional file 4: Table S1). The culture numbers, total body weight and intestinal contents of grass carp and crucian carp were higher than those of the other species in the culture ponds (Table 1).

**Table 1:** Body parameters of sampled fish and the yield proportion of co-cultured species in the farm

	Grass carp	Common carp	Silver carp	Crucian carp	Bighead carp
body weight (g)^a^	902 ± 286	864 ± 251	1158 ± 350	299 ± 103	988 ± 277
intestinal content (g)^a^	25.9 ± 20.8	7.2 ± 7.1	25.4 ± 18.3	4.4 ± 3.4	19.9 ± 13.9
culture number (ind)^b^	121,000	26,000	10,000	332,000	9000

^a^Determined based on the sampled fish

^b^The numbers of cultured fish species in the farm in 2020, which were calculated during the harvest season

**Fig. 1:**
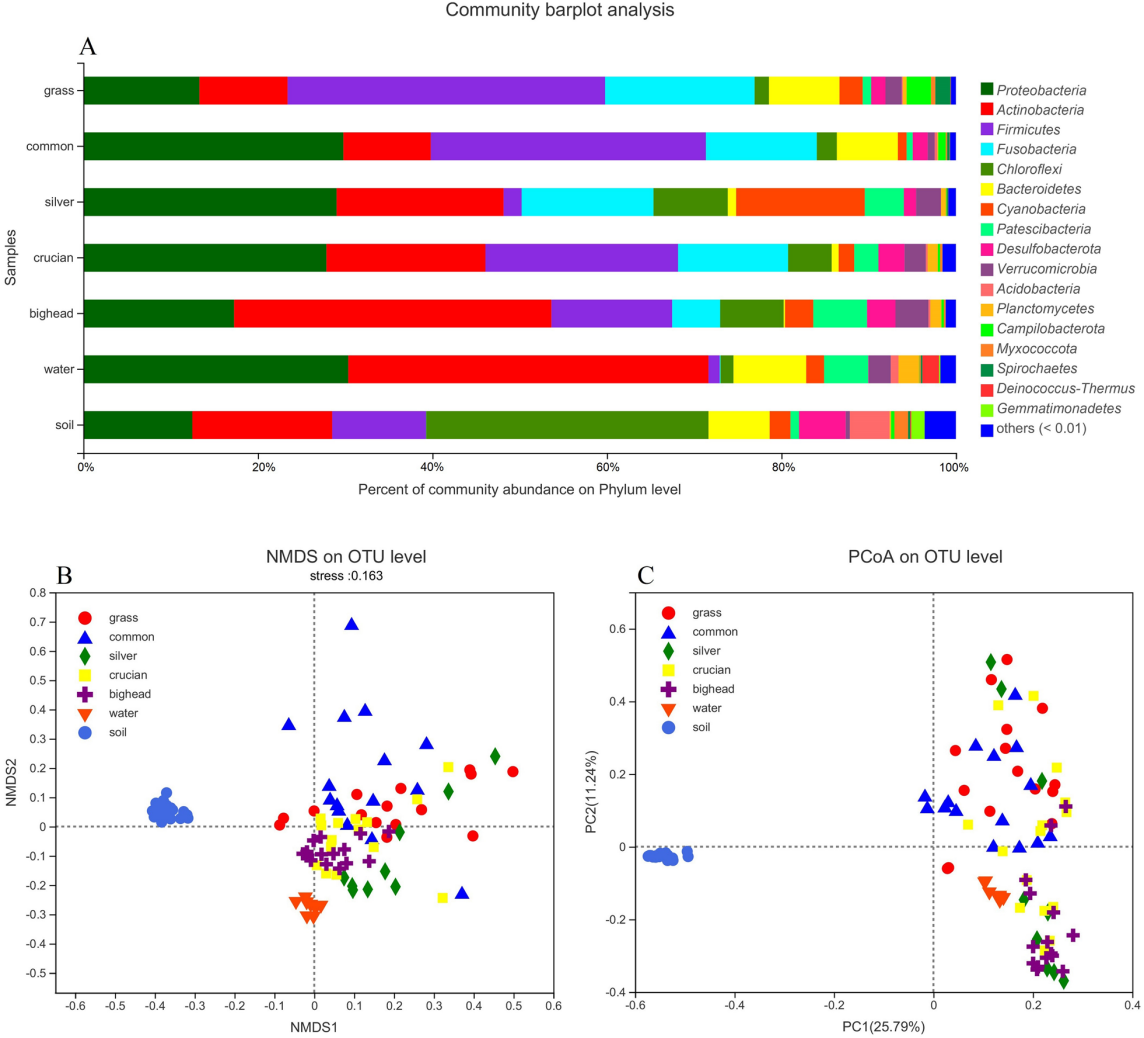
**A** Bacterial community structure at the phylum level for sample groups. Non-metric multidimensional scaling analysis **B** and principal co-ordinates analysis **C** revealed significant variations between intestinal and soil bacterial communities. Grass, common, silver, crucian, and bighead indicating intestinal bacterial communities in the five analyzed carp species; water and soil indicating planktonic and soil bacterial communities

After Illumina Miseq sequencing and quality controlling, 5.3 million clean sequences with an average length of 413 base pairs were obtained. After removing chloroplast sequences and conducting normalization, 27,084 sequences from each sample were used in the subsequent analysis. From the 101 libraries, a total of 13,937 OTUs and 2036 genera were obtained. Good’s coverage values of all libraries were higher than 94.5%. The dominant bacterial phyla in planktonic samples were *Actinobacteria* and *Proteobacteria*, with relative abundances of 41.3% and 30.4%, respectively. *Proteobacteria* (23.0%), *Firmicutes* (22.9%), *Actinobacteria* (17.8%), and *Fusobacteria* (12.4%) dominated intestinal samples, and *Chloroflexi* (32.4%), *Actinobacteria* (16.0%), *Proteobacteria* (12.5%), and *Firmicutes* (10.7%) dominated soil samples (Fig. 1A). The OTU table is provided in Supporting Information Additional file 5: Table S2 and the top 50 bacterial genera are provided in Additional file 2: Figure S2.

Student's *t*-test analysis of Shannon, Simpson, Ace, and Chao indexes revealed higher diversity of the four indexes was found in soil samples than intestinal and planktonic samples (P < 0.001, the results of *t*-test are provided in Additional file 4: Table S1). Non-metric multidimensional scaling analysis revealed significant variations in beta diversity between intestinal, water, and soil samples, and dispersion was greater in intestinal samples than in water and soil samples (Fig. 1B) and the principal co-ordinates analysis also revealed significant variations between intestinal and soil samples (Fig. 1C). Analysis of similarity also revealed significant variations between intestinal and planktonic samples (R = 0.3417, P = 0.002), between planktonic and soil samples (R = 1, P = 0.001), and between intestinal and soil samples (R = 0.7908, P = 0.001). Furthermore, significant variations of beta diversity were found among the five fish species (R = 0.3231, P = 0.001), and soil bacterial communities were more similar with intestinal bacterial communities in common carp (R = 0.9201, P = 0.001) in comparison to grass carp (R = 0.9757, P = 0.001), silver carp (R = 0.9771, P = 0.001), crucian carp (R = 0.9808, P = 0.001) and bighead carp (R = 1, P = 0.001).

### Core genera

**Fig. 2:**
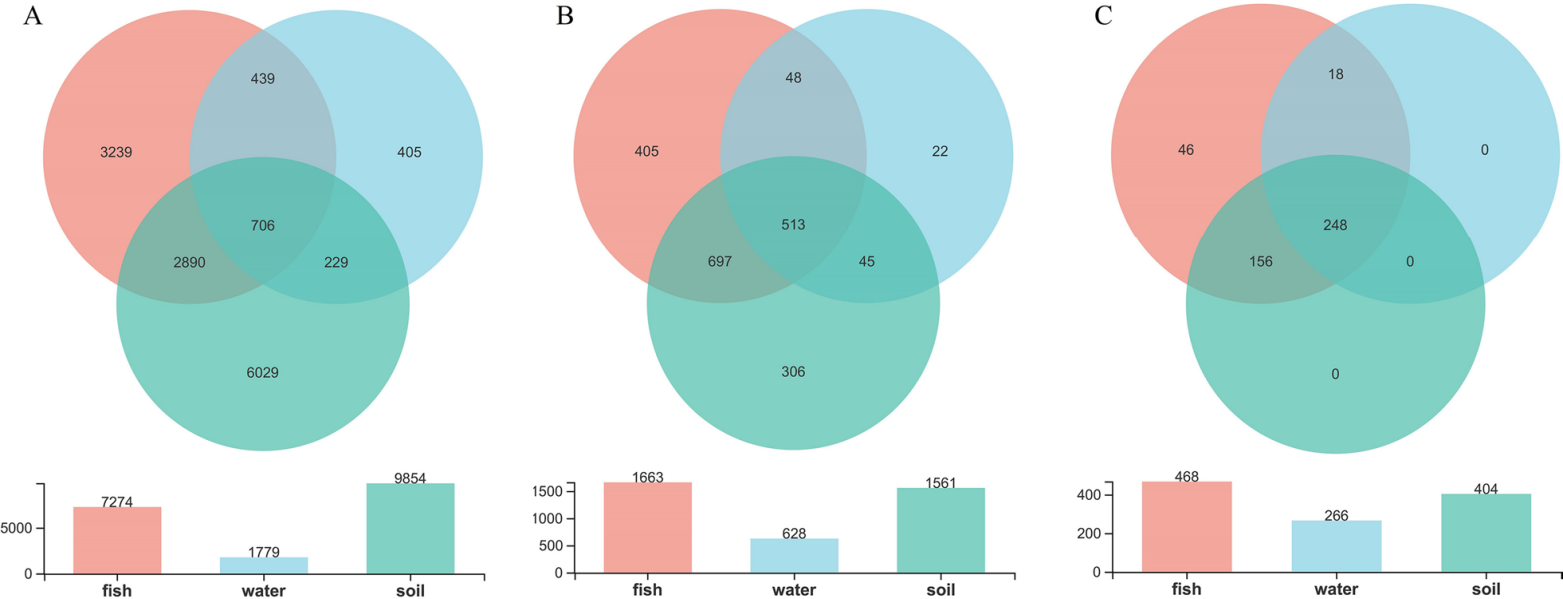
Venn diagrams showing the number of shared bacterial OTUs **A** and genera **B** in planktonic, intestinal and soil bacterial communities, and that the shared intestinal bacterial genera by the five carp species were also found in soil **C**. Fish, water and soil indicating intestinal, planktonic and soil bacterial communities

The Venn diagram showed that 3596 OTUs were shared by intestinal and soil samples (Fig. 2A). Among them, 706 core OTUs were shared by intestinal, planktonic, and soil bacterial communities. Another 2890 OTUs were shared by only intestinal and soil samples, indicating that some bacterial OTUs were attached to particulate matter in the water and were filtered by the 5-μm pore filters. In total, 3596 OTUs shared by intestinal and soil bacterial communities accounted for 49.4% and 36.5% of OTUs in intestinal and soil samples. At the genus level, 513 genera were shared by intestinal, planktonic, and soil bacteria, and another 697 genera were shared by only intestinal and soil samples. In total, 1210 genera shared by intestinal and soil bacteria accounted for 72.8% and 77.5% of genera in intestinal and soil samples (Fig. 2B).

The Venn diagram also showed that 78.7%, 73.6%, 83.9%, 78.1%, and 85.6% of intestinal genera in grass carp, common carp, silver carp, crucian carp, and bighead carp were shared with soil bacterial communities, representing 58.9%, 61.7%, 33.5%, 53.8%, and 47.8% of soil genera (Additional file 3: Figure S3). The results indicated that more soil bacterial genera were shared by intestinal bacterial communities in common and grass carp. In addition, 468 genera were shared by all five fish species and 404 of them were also found in soil (Fig. 2C).

### Soil bacterial phyla

**Fig. 3:**
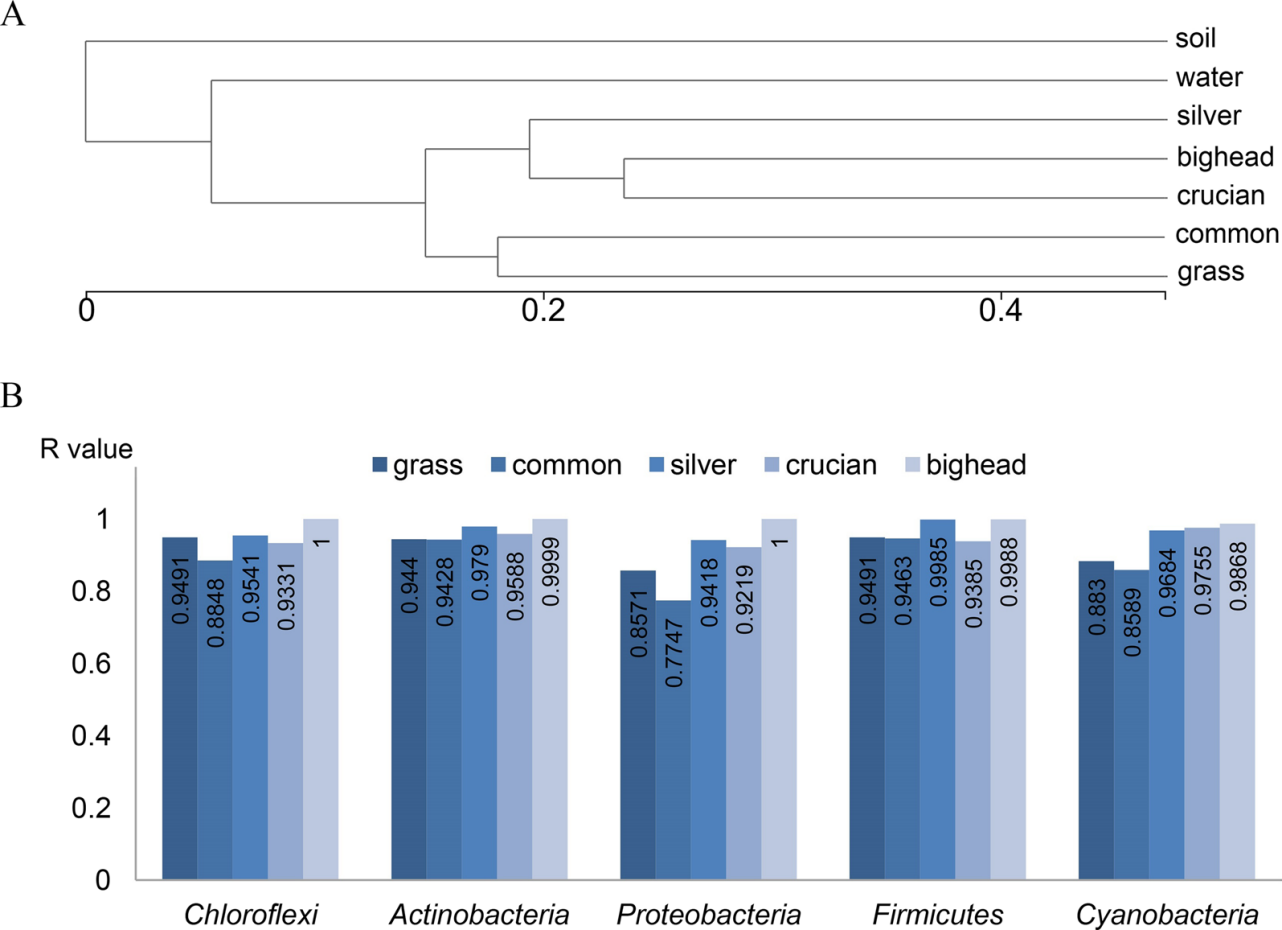
**A** Hierarchical clustering results showing the Bray–Curtis distances between sample groups. **B** The results from analysis of similarity showing the Bray–Curtis distances of dominant bacterial phyla between soil and intestinal bacterial communities in each of five analyzed carp species (P < 0.01). Grass, common, silver, crucian, and bighead indicating intestinal bacterial communities in the five analyzed carp species; water and soil indicating planktonic and soil bacterial communities

The hierarchical clustering results indicated that bacterial communities in soil were more similar to that in common and grass carp than in the other species (Fig. 3A), in accordance with the comparison of Bray–Curtis distances and shared bacterial genera. For further analysis of the dominant phyla in soil, sequences allocated to *Chloroflexi*, *Actinobacteria*, *Proteobacteria*, and *Firmicutes* were retrieved from obtained libraries in this study. As an important bacterial phylum in the bacteria-plant interactions (Bahareh et al. 2021), *Cyanobacteria* communities were also compared.

In this study, *Chloroflexi* was the top bacterial phylum in the soil and significantly higher relative abundances of *Chloroflexi* were found in the soil than in the intestine samples. The analyses of similarity showed that soil *Chloroflexi* communities were more similar to that in common carp, crucian carp and grass carp than other carp species and *Firmicutes* communities in crucian carp, common carp and grass carp were more similar to that in soil. In addition, *Actinobacteria* and *Proteobacteria* communities in common carp and crucian carp possessed smaller Bray–Curtis distances with soil bacterial communities (Fig. 3B).

The sequences associated with Cyanobacteria were also retrieved. Helch's *t*-test analysis revealed no significant differences of *Cyanobacteria* abundances among planktonic, soil, and intestinal bacteria, but *Cyanobacteria* were more abundant in the intestinal bacterial communities in silver carp compared to the other four fish species (P < 0.001). The analyses indicated that soil *Cyanobacteria* communities were more similar to that in common carp and grass carp (Fig. 3B).

### Source tracking

The SourceTracker analysis results showed the contributions of intestinal bacterial communities to soil (Table 2). In the soil bacterial community in rice field, an average of 9.9% of bacterial genera was confirmed from intestinal bacterial communities. The source of 3.6% of soil bacterial genera was the bighead carp intestines and 3.0% was from the crucian carp intestines. In grass carp, common carp and silver carp, intestinal bacterial communities contributed lesser bacterial genera. An average of 90.3% of soil bacterial genera was from unknown sources. Comparing between the analyzed bacterial phyla, bighead carp contributed more *Cyanobacteria* genera and other carp species contributed more *Chloroflexi* genera than other bacterial phyla. Between five carp species, grass carp contributed more *Chloroflexi* genera and crucian carp made more contribution of *Actinobacteria* and *Firmicutes* genera to soil bacterial communities (Table 2).

**Table 2:** SourceTracker results showing the contributions of intestinal bacteria in each carp species to soil bacterial genera, on the whole (bacteria) and on dominant bacterial phyla

	Bighead carp %	Common carp %	Crucian carp %	Grass carp %	Silver carp %	Unknown %
bacteria	3.6 ± 0.9	1.0 ± 0.2	3.0 ± 0.8	1.9 ± 0.4	0.5 ± 0.5	90.3 ± 1.7
*Chloroflexi*	2.3 ± 0.7	2.6 ± 0.7	2.4 ± 0.8	3.8 ± 0.8	1.5 ± 0.5	87.4 ± 2.7
*Actinobacteria*	0.5 ± 0.5	1.1 ± 0.3	1.5 ± 0.7	1.2 ± 0.4	0.9 ± 0.5	95.0 ± 1.8
*Proteobacteria*	1.0 ± 0.2	0.9 ± 0.3	1.0 ± 0.3	1.0 ± 0.0	0.4 ± 0.5	95.8 ± 0.8
*Firmicutes*	0.9 ± 0.3	0.0 ± 0.2	1.3 ± 0.6	0.9 ± 0.3	0.3 ± 0.5	96.3 ± 0.9
*Cyanobacteria*	3.0 ± 2.4	0.9 ± 0.8	1.0 ± 0.7	0.7 ± 0.6	0.7 ± 0.6	93.8 ± 4.2

The mean values and standard deviations were obtained from 24 soil samples

## Discussion

### Soil bacterial communities

Rice is an important crop species with a huge cultivated area, mainly in Asia. Traditionally, rice cultivation requires a huge amount of water. In addition, aquaculture produces wastewater that must be purified before discharge. One strategy to address these issues is to blend aquaculture operations and rice cultivation. Using wastewater from aquaculture ponds (Van Tung et al. 2021) or integrating aquaculture of suitable species in rice fields (Li et al. 2021b) improves the utilization rate of water and reduces wastewater discharge rates. Consequently, these methods have been promoted in some regions, especially in arid and semi-arid regions.

Zhao et al. (2021) reported that the soil bacterial community structure in a rice-fish system was obviously different from that of the traditional rice field, and Chen et al. (2017) detected higher bacterial community diversity in soil irrigated with aquaculture wastewater than in those irrigated with lake water. However, Li et al. (2021b) reported that the high amounts of protein entering agricultural soil via pellet feed and fish feces lowered soil bacterial diversity in a rice-fish system. In the current study, we found that bacterial diversity in rice field soil irrigated with aquaculture wastewater was lower than that reported by Chen et al. (2017) and Zhao et al. (2021), but it was higher than that of intestinal bacterial communities in the cultured fish. To date, analysis of the relationship between intestinal bacteria and soil bacteria in rice-fish systems are rare. Our results showed that 36.5% of soil OTUs and 77.5% of bacterial genera were shared by cultured carps. This result indicates that intestinal bacteria probably impact soil bacterial communities through irrigation, and that some bacterial species from the fish intestine may colonize the soil. Xiao et al. (2022) reported that microbial inoculation had a significant impact on soil bacterial community structure and an even more significant impact on rare bacteria than on dominant bacteria.

### Intestinal bacterial communities

Many factors can impact intestinal bacteria, including food, drugs, and environmental conditions. Some studies have shown that most intestinal bacterial OTUs in aquatic animals are also found in the sediment and water, indicating that the intestinal bacteria in aquatic animals are derived from the environment (Sun et al. 2020). However, research has also indicated that intestinal bacterial communities are host-specific, even when various species live together in the same environment, e.g., in wild Asian carp (Li et al. 2018), in sole (*Solea senegalensis*) and turbot (*Scophthalmus maximus*) (Martins et al. 2013). In a study of grass carp, crucian carp, and bighead carp co-raised in aquaculture ponds, Li et al. (2015) found higher intestinal microbial diversity in the filter feeding bighead carp than in the other two species, which suggested that the intestinal microbiota assemblage resulted from species-specific selective pressures and was not a direct duplicate of the microbial community in the environment. In the present study, the dominant bacterial phyla in the five carp species differed somewhat from those reported in previous studies. *Fusobacteria* was previously reported to be the most dominant phylum in cultured grass carp, crucian carp, and bighead carp (Li et al. 2015) and in common carp (Yu et al. 2021). Intestinal microbiota structure in perch (*Perca fluviatilis*) was obviously impacted by food rationing and predator presence, as *Fusobacteria* abundances increased under low food rations and predation stress (Zha et al. 2018). In the present study, *Proteobacteria* was the most dominant phylum, as was reported in previous studies of wild silver carp, bighead carp, grass carp, and common carp (Li et al. 2018). We recognized that variations of intestinal bacterial communities exist between species and individuals, so we used a large sample size from a rice-fish system with the long-term irrigation using aquaculture wastewater and focused on comparing soil bacterial communities and intestinal bacterial communities in each of five co-cultured fish species.

### The co-culture of carp species

The co-culture of carp species is common in some Asian regions. Based on the mentioned reports, we recognized that aquaculture wastewater impacts the soil bacterial communities. However, it is relatively difficult to compare the impacts of fish species in the co-culture model, in comparison to the mono-culturing these fish species and irrigating rice field separately. The aquaculture operations in the co-culture and mono-culture modes were different, and the co-culture of these species optimized the use of feed and space, lowered the cost and increased the production. Under the co-culture model, phytoplankton is usually cultivated in aquaculture ponds to lower the pollutant level, particularly ammonia which is well known to have the severe biological toxicity to aquatic animals. Phytoplankton breeds zooplankton. Silver carp and bighead carp are routinely cultured to control the phytoplankton and zooplankton levels in these aquaculture ponds. Other carp species usually live on the artificial compound feeds. In this study, our results showed that soil bacterial communities were more similar to that in common carp and grass carp intestines than the other three carp species, on the whole or on most of dominant bacterial phyla.

In carp aquaculture, the co-cultured species and their proportions differ among different regions and even within farms depending on culture models and market requirements. In the region where the present study was conducted, grass carp and crucian carp are the main culture species, but common carp are also popular with local consumers. Silver carp and bighead carp are popular in many regions of south and east China, but they play minor roles in the co-culture ponds of northwest China. The co-culture model used in the farm evaluated in this study is common in northwest China. In this study, the intestinal and soil bacterial communities were obviously different. Many factors impact the soil bacterial communities, and aquaculture operations impact environments in many ways, such as fish mucus (Molina and Fernandez 2020). Fish feces are merely one factor. The impacts of intestinal bacteria on soil bacterial communities under different co-cultured models remain yet unclear. In this system, the similarity between intestinal and soil bacterial communities was high for common carp and relatively low for crucian carp, indicating that intestinal bacteria in the numerically dominant fish species were not always more similar to the rice field soil bacteria than those of less abundant carp species.

### Source tracking

*Cyanobacteria* represent one of the earliest branches of biological evolution on Earth, and they have been subjected to various selective pressures over time (Esteves-Ferreira et al. 2018). The interactions between *Cyanobacteria* and plants have occurred in different ways and at different levels and have been both beneficial of harmful. In recent years, interest in *Cyanobacteria*-plant interactions has grown, especially in rice-growing areas where the most efficient nitrogen-fixing *Cyanobacteria* are present (Bahareh et al. 2021). In this study, no significant differences in *Cyanobacteria* abundances were found between intestinal and soil bacteria. Members of another phylum, *Chloroflexi*, were found to be dominant in rice field soil, especially at the mature stage (Sohn et al. 2016), and in soil from rice-crab co-culture fields (Jiang et al. 2021). In our study, *Chloroflexi* was the top phylum in rice field soil, and the relative abundances were significantly higher in the soil than in fish intestines. Furthermore, the community structures of most analyzed soil bacterial phyla were more similar to that in common carp and grass carp, but soil *Firmicutes* communities were more similar to that in crucian carp. These results suggest that certain intestinal bacterial phyla in one fish species may be more similar to that in soil than other bacterial phyla.

However, there is a distance between the bacterial community similarity and the potential impacts. SourceTracker is a Bayesian approach which can be used to estimate the proportion of contaminants and possible source environments (Knights et al. 2011) and proved highly effective at predicting the composition of known sources (Brown et al. 2019; McGhee et al. 2020). Using the SourceTracker approach, Zhou et al. (2021) found that the sediment was a more important source of bacteria to the shrimp gut than the pond water and Sun et al. (2021) found that manure and original soil were the main source of the microbiome and resistome of the surface soil and rhizosphere soil. Our SourceTracker result revealed that a low contribution of intestinal bacterial communities to soil although the high proportion of shared bacterial genera, indicating intestinal bacteria were not the main sources of soil bacterial communities. In addition, the result also showed that bighead carp and crucian carp showed a relatively higher effect on soil bacterial communities than other carp species, indicating that the numerically dominant carp species do not necessarily impact soil more significantly. The analysis results on certain intestinal bacterial phyla also showed that one fish species may impact soil bacterial communities more significantly in some bacterial taxa than others.

In conclusion, although a high proportion of bacterial genera shared by intestinal and soil bacterial communities, the results from analysis of similarity and SourceTracker analysis showed that a low similarity and contribution of intestinal bacterial communities in co-cultured carps to soil communities in rice fields irrigated with aquaculture wastewater. Our comparison of intestinal bacterial communities in each carp species and soil bacterial communities also indicated that the dominant fish species in the co-cultured system did not necessarily have a more significant impact on the soil bacterial communities than less abundant fish species. Moreover, intestinal bacterial communities in a single fish species impacted certain soil bacterial phyla more significantly than others.

## Supplementary Information

**Additional file 1:** **Figure S1.** Location of sampling sites.

**Additional file 2:** **Figure S2.** Community heatmap showing the top 50 genera.

**Additional file 3:** **Figure S3.** Venn diagrams showing the number of shared bacterial genera by soil and intestinal bacterial communities in each carp species.

**Additional file 4:** **Table S1.** Sample information and alpha diversity indexes.

**Additional file 5: Table S2.** The OTU table.

## Data Availability

The data that support the findings of this study are available in the supplementary information of this article. The raw gene sequences obtained in this study have been submitted to the NCBI SRA database (https://submit.ncbi.nlm.nih.gov/subs/sra/) under the accession number PRJNA741343.
