# Supplementary figures and images for: Bacterial communities in co-cultured fish intestines and rice field soil irrigated with aquaculture wastewater
Source: AMB Express. 2022 Oct 22;12:132. doi: 10.1186/s13568-022-01475-x (PMC9588148; doi:10.1186/s13568-022-01475-x)

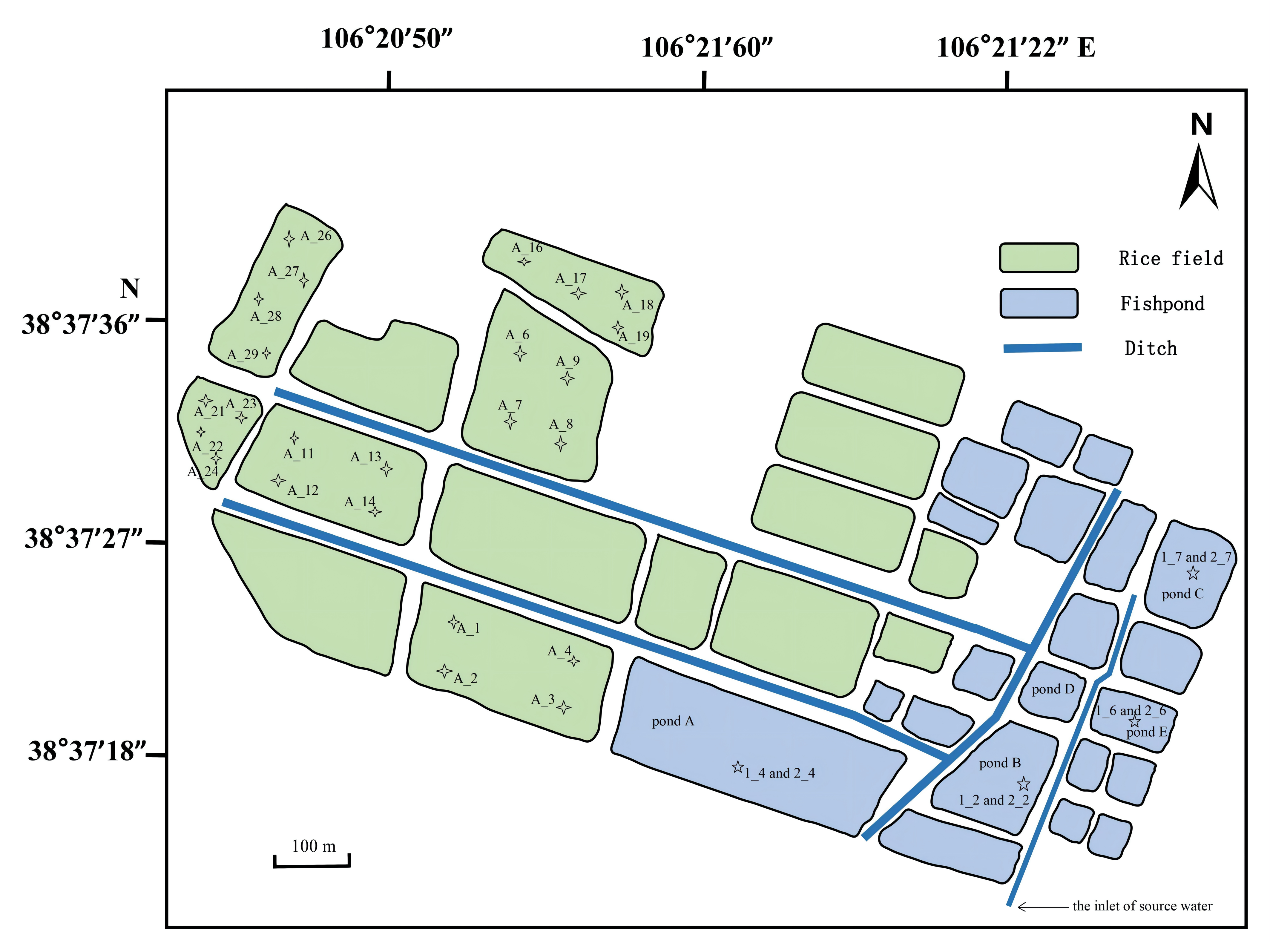

Supplement: Supplementary file 1 — Additional file 1: Figure S1. Location of sampling sites. [file 13568_2022_1475_MOESM1_ESM.tif]

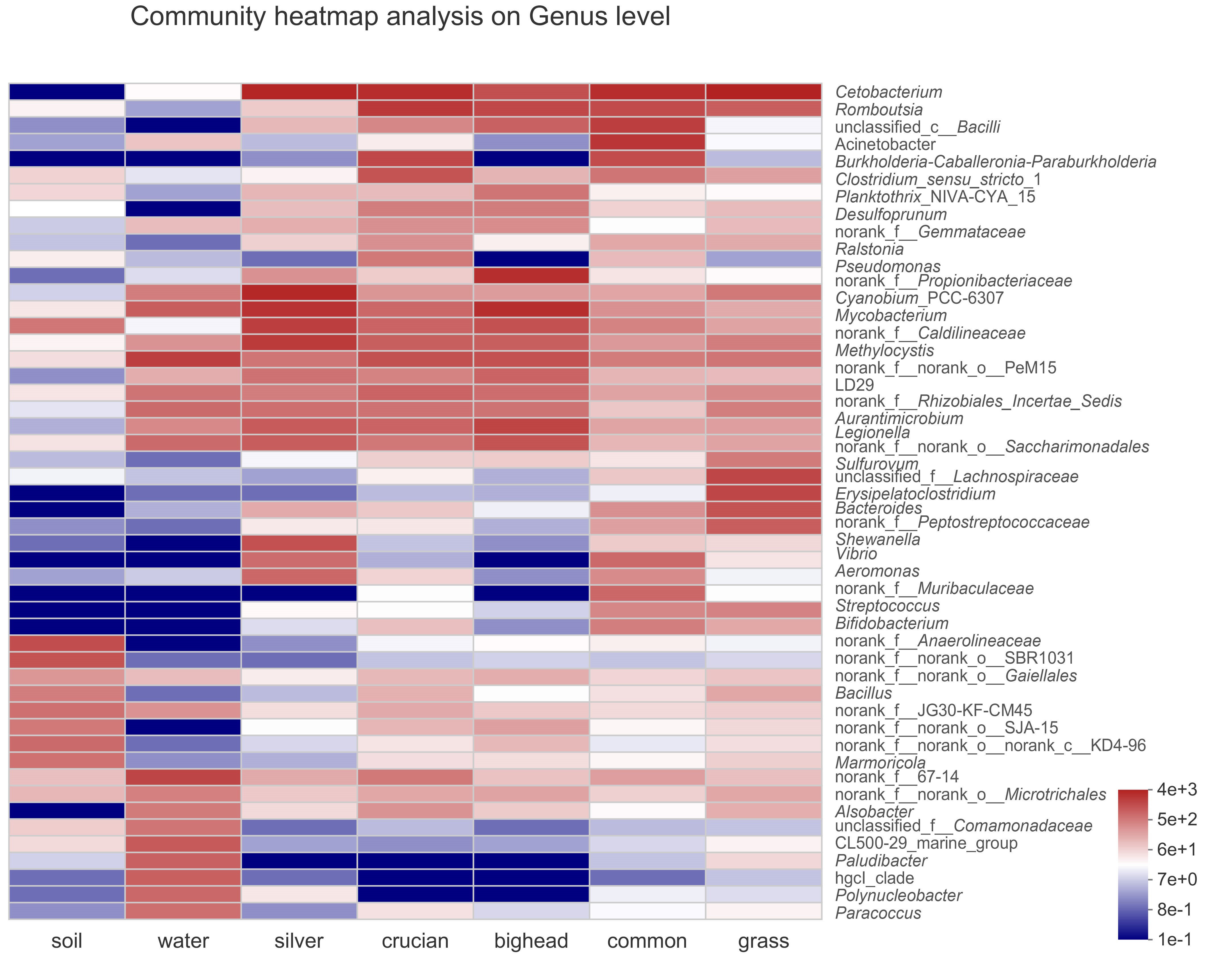

Supplement: Supplementary file 2 — Additional file 2: Figure S2. Community heatmap showing the top 50 genera. [file 13568_2022_1475_MOESM2_ESM.jpg]

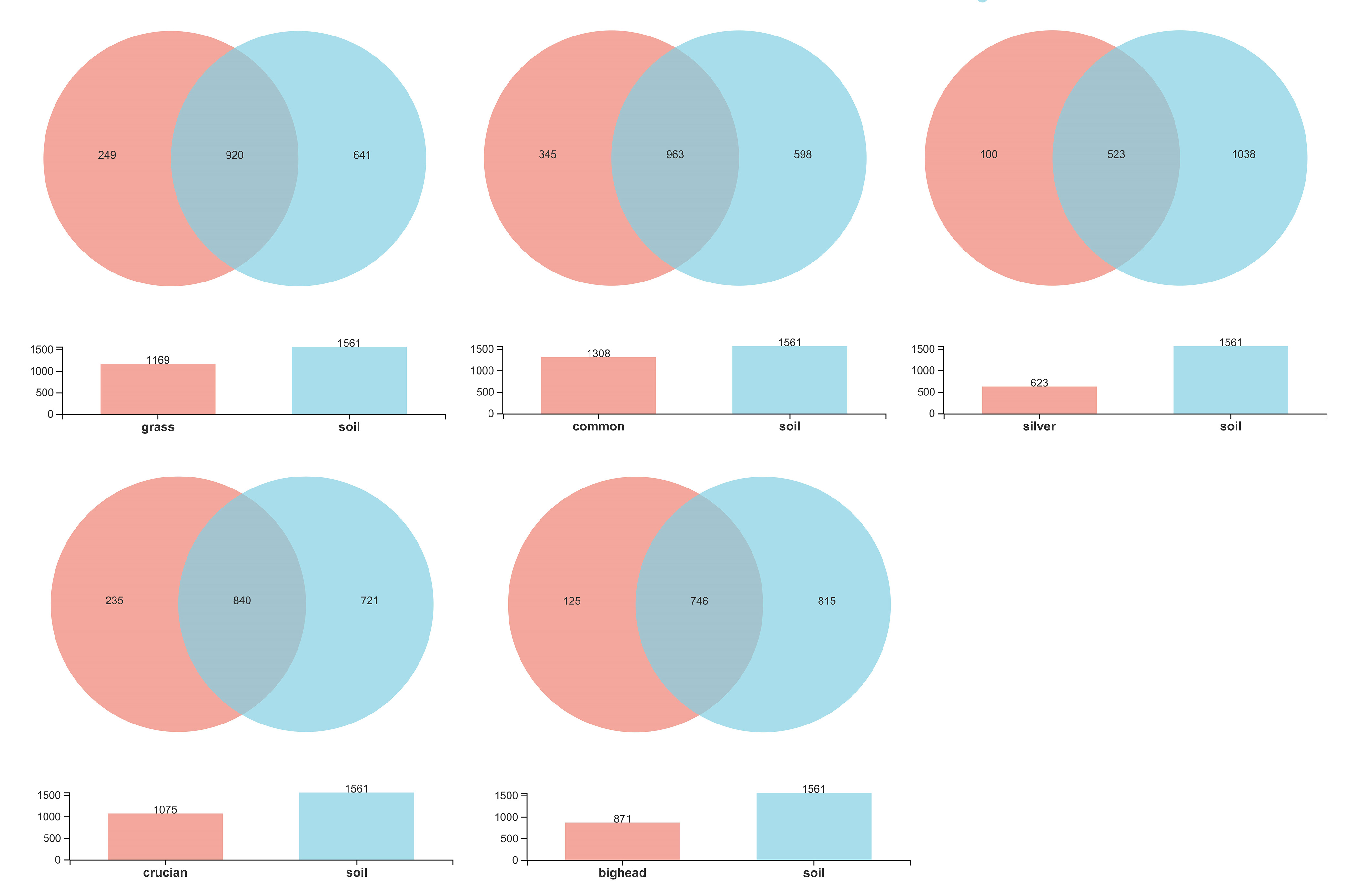

Supplement: Supplementary file 3 — Additional file 3: Figure S3. Venn diagrams showing the number of shared bacterial genera by soil and intestinal bacterial communities in each carp species. [file 13568_2022_1475_MOESM3_ESM.jpg]
